# ﻿*Lithocarpustapanuliensis* (Fagaceae), a new stone oak from northern Sumatra and its role as an important resource for critically endangered orangutans

**DOI:** 10.3897/phytokeys.234.106015

**Published:** 2023-10-20

**Authors:** Try Surya Harapan, Wei Harn Tan, Thoriq Alfath Febriamansyah, Nurainas, Syamsuardi, Joeri Sergej Strijk

**Affiliations:** 1 Southeast Asia Biodiversity Research Institute, Chinese Academy of Sciences & Center for Integrative Conservation, Xishuangbanna Tropical Botanical Garden, Chinese Academy of Sciences, Mengla, Yunnan 666303, China Xishuangbanna Tropical Botanical Garden, Chinese Academy of Sciences Mengla China; 2 Yunnan International Joint Laboratory of Southeast Asia Biodiversity Conservation & Yunnan Key Laboratory for Conservation of Tropical Rainforests and Asian Elephants, Menglun, Mengla, Yunnan, 666303, China Universitas Andalas Padang Indonesia; 3 Herbarium Andalas, Department of Biology, Faculty of Mathematics and Natural Sciences, Universitas Andalas, Jl. Universitas Andalas, Limau Manis, Padang 25163, West Sumatra, Indonesia Yunnan International Joint Laboratory of Southeast Asia Biodiversity Conservation & Yunnan Key Laboratory for Conservation of Tropical Rainforests and Asian Elephants Mengla China; 4 Faculty of Science, Universiti Brunei Darussalam, Jalan Tungku Link, Gadong BE1410, Darussalam, Brunei Universiti Brunei Darussalam Gadong Brunei; 5 Alliance for Conservation Tree Genomics, Pha Tad Ke Botanical Garden, 06000 Luang Prabang, Laos Alliance for Conservation Tree Genomics, Pha Tad Ke Botanical Garden Luang Prabang Laos

**Keywords:** Batang Toru, Hoteng, *Lithocarpustapanuliensis*, Sumatran Fagaceae, Tapanuli orangutan, food habits

## Abstract

A new species of stone oak, *Lithocarpustapanuliensis* Harapan, W.H.Tan, Nurainas & Strijk from South Tapanuli, North Sumatra, Indonesia is described. We provide colour photographs, a distribution map and a new IUCN conservation status assessment for inclusion on the global Red List. The unique cupule morphology, particularly the shape, placement and distinctness of the cupule protuberances, are distinctive from other *Lithocarpus* species in the region. Ecological interactions (e.g. consumption and nesting) with Tapanuli orangutans were recorded in the field.

## ﻿Introduction

The tropical rainforest of Sundaland is one of the most megadiverse regions on the Planet (Myers et al. 2000), with the island of Sumatra as one of the larger remaining land masses in this submerged continental shelf. Sumatra serves as a refugium for many Sundaic flora and fauna species (Woodruff 2010). Some of the world’s most critically endangered megafauna (Sumatran elephant (*Elephasmaximussumatranus* Temminck, 1847); Sumatran rhinoceros (*Dicerorhinussumatrensis* Fischer, 1814); Sunda tiger (*Pantheratigrissondaica* Temminck, 1844) and Sumatran and Tapanuli orangutans ((*Pongoabelii* Lesson, 1827) and (*Pongotapanuliensis* Nurcahyo, Meijaard, Nowak, Fredriksson & Groves, 2017)) are found here. Sumatra is also botanically diverse, with an estimated number of 10,600 plant species and more than 300 endemics (Roos et al. 2004). Moreover, the plant diversity of Sumatra is hypothesised to be as diverse as Borneo and much richer than the other neighbouring islands of Java and Sulawesi (Meijer 1981). However, floristic studies in Sumatra have been neglected in the past, with the mistaken assumption of being well documented due to its similarity with flora from the Malay Peninsula (Whitten 1984; Laumonier 1997). Recent discoveries in various taxa prove otherwise, sparking renewed interest in the island’s rich untapped diversity.

*Lithocarpus* Blume (Stone oaks) is the second largest genus in the Fagaceae family (Camus 1952–1954), with approximately 347 species recorded globally, including 32 found in Sumatra, of which five species are endemic to the island (POWO 2023; Strijk 2023). Species from this genus are commonly found throughout Sumatra, inhabiting many different attitudinal habitats, from lowland to montane forest (Laumonier 1997; Fujii et al. 2006). Fujii et al. (2006) found a great species diversity of *Lithocarpus* in Sumatra between 400 and 700 m above sea level, with several species having distributions limited to certain elevations. Along with several families like Lauraceae and Myrtaceae, Fagaceae are a major component of the lower tropical montane rainforest between 900 and1500 m above sea level (Cockburn 1972; Laumonier 1997). Species such as *Lithocarpuspallidus* (Blume) Rehder and *Lithocarpuspseudomoluccus* (Blume) Rehder often constitute the canopy layer of submontane forest in Sumatra (Laumonier 1997). Eight species of *Lithocarpus* were also recorded in Sumatra’s upper montane forest (1400–2500 m a.s.l.) by Fujii et al. (2006), further highlighting the flexibility of the genus in occupying different ecological niches.

South Tapanuli is one of the three forest blocks that make up the Batang Toru Ecosystem and is the last refuge for the recently described, Critically Endangered and extremely rare Tapanuli orangutans (Kuswanda et al. 2020). The land cover within the Batang Toru Ecosystem consists of a mosaic of mixed plantations and primary and secondary forests (Meylia and Mustari 2022). During a field survey conducted in South Tapanuli in February 2023, specimens of an unknown *Lithocarpus* were discovered. Further morphological comparisons with other relatives in Malesia clearly distinguish it as a new species due to its distinctive cupule morphology (Cockburn 1972; Soepadmo 2000; Phengklai 2008). Hence, we describe and name it as *Lithocarpustapanuliensis*, providing a description, accompanied by photographs and a morphological comparison with closely-related species, as well as an exploration of its interactions with Tapanuli orangutans.

## ﻿Taxonomy

### ﻿ Lithocarpus tapanuliensis

Taxon classificationPlantaeFagalesFagaceae

Harapan, W.H.Tan, Nurainas & Strijk sp. nov.

ECB1181E-5D32-500B-A788-DD57ECB1AB3B

urn:lsid:ipni.org:names:77329008-1

Fig. 1

#### Type material.

***Holotype*.** Indonesia, North Sumatra Province, South Tapanuli Regency, Sipirok District, Bulu Mario Village, Pilar Forest (Fig. 2). 1°34'53.9"N, 099°11'38.2"E, elevation 894 m, 23 February 2023, ***Holotype***: ANDA [ANDA00000051794]; Isotypes: ANDA [ANDA00000051793].

#### Diagnosis.

*Lithocarpustapanuliensis* distinguishes itself from similar species through its presence and placement of unique bullate protuberances covering the cupule and the distinct presence of a narrow ring of small denticulated plates around the rim. It differs from *L.elegans* (Blume) Hatus. ex Soepadmo with tiny, pointed scale-like appendages, *L.confragosus* (King ex Hook.f.) A.Camus with close-set warts, *L.corneus* (Lour.) Rehder with the diamond-like pattern and *L.pulcher* (King) Markgr. with tuberculate cupules. The cupule of *L.tapanuliensis* covers almost 3/5 of the nut (in contrast with *L.pulcher* and *L.confragosus*, whose cupule encloses almost the entire nut). The surface of the cupule is slightly tomentose and dark brown with distinct protuberances (whereas *L.confragosus*, *L.corneus* and *L.pulcher* lack such because of the absence of lamellae) (Table 1).

**Table 1.** Morphological differences between Lithocarpustapanuliensis sp.nov and other species of Lithocarpus in the surrounding region from literature (Cockburn 1972; Soepadmo 2000; Phengklai 2008).

Characters	*Lithocarpustapanuliensis* Harapan, W.H. Tan, Nurainas & Strijk	*L.confragosus* (King ex Hook.f.) A.Camus	*L.corneus* (Lour.) Rehder	*L.luteus* Soepadmo	*L.elegans* (Blume) Hatus. ex Soepadmo	*L.pulcher* (King) Markgr.
1. Cupule surface	Up to ⅘ covered with bullate protuberances; upper ⅕ with narrow ring of small denticulated plates. The surface of the cupule is slightly tomentose	Outside irregularly set with rounded to pointed short tubercles.	Outside with triangular to rhomboid bracts, the centre and margin ridged or fused with cupule and ± united into concentric rings.	Woody, tomentose, lamellate; thick, hairy, enclosing up to half of the acorn; lamellae obscure or slightly distinct, edge denticulate, set in 8–10 regular lines.	Adpressed tomentose, scale-like appendages distinct, appressed, woody imbricate, set in regular lines.	Woody, tomentose, covered in distinct sturdy tuberculate, irregularly and densely set on the upper part of cupule, spreading out towards the base.
2. Nut scar	Concave.	Flat to concave, basal only.	Scar covering ½ to most of nut, convex.	Flat.	Flat to concave, basal only.	Scar covering ¾ of the nut, deeply convex.
3. Size of acorns (*l* × *w*)	1.9–2.9 cm long, 2.6–3.4 cm in diam.	1.5–2.5 cm long, 2–4 cm in diam.	2.5–3.4 cm long, 3.3–4.9 cm in diam.	1–1.5 cm long, 2–2.5 cm in diam.	1.5–2.5 cm in length, 1.5–3 cm in diam.	2–4 cm long, 4–5 cm in diam.
4. Acorn position	Sessile, solitary along the rachis and spaced.	Sessile or with stalk up to 1 cm.	Sessile, singular or in 2s, 3s or 4s.	Sessile, solitary or more common in clusters.	Sessile or with stalk up to 0.5 cm, solitary.	Sessile, solitary along the rachis.
5. Nut surface	Sparsely tomentose.	Glabrous, smooth.	Tomentose around the apex.	Densely fulvous to greyish-tomentose.	Glabrous, smooth.	Sparsely tomentose, brown.
6. Wall; nut covering extent of the cupule.	Free from the cupule; up to half of the nut covered.	For the greater part free from the cupule; enclosing greater part of the nut,except for opening.	Free from the cupule; enclosing ca.½ of the nut.	Free from the cupule; enclosing ca. ⅓ of the nut.	Free from the cupule; enclosing ca. ½ nut.	Mostly adnate to the cupule; enclosing greater part of the nut except for opening.
7. Nut shape	Obovoid (more flat at the apex)	Depressed, ovoid-globose, top rounded and depressed umbonate at the centre, base truncate	Subglobose to turbinate, apex rounded, flat, or slightly concave	Ovoid to sub hemispherical	Ovoid or depressed ovoid to subglobose, apex rounded	Obconical- hemispherical. Top flat or convex. Base deeply convex
8. Leaf shape; size (*l* × *w*)	Elliptic to oblong, (14) 16–17(20) × (4.7)6–7(8.5) cm, margin entire, apex cuspidate, base attenuate.	Elliptic to oblong, to broadly elliptic, (10–)12–18(–27) × (3.5–)5–7(–10) cm, broadest around the middle line.	Elliptic to oblong, (5–)10–15 × 2–4.5 cm. Base cuneate to subrounded and symmetric or oblique, margin dentate, shallowly undulate, or rarely entire, apex acuminate to acute.	Elliptic to obovate, (7–)9–12 (–15) × (2.5 –) 3.5–5 (–6) cm, broadest at or slightly below the middle, base acute to cuneate, top acute to 1 cm acuminate.	Narrowly to broadly obovate or elliptic, (9–)12–17(–21) × 3–6(–8) cm base acute or cuneate, margin revolute, apex bluntly acute or acuminate.	Broadly elliptic to oblong, (10–) 15–20(–30) × (4–) 6–8(–12.5) cm, base acute to cuneate, margin revolute, apex acute to acuminate.

#### Description.

A large tree without buttresses, up to 35 m tall. ***Bark*** rough, lightly fissured, greyish-green with whitish lenticel. Inner bark is dark red forming longitudinal slits. ***Twigs*** diameter 0.2–0.4 cm, smooth, striate, bud imbricate 0.5 mm. ***Branches*** dark brown. ***Leaves*** simple, underneath tomentose, dark green above and fawn green below when fresh; above, dull greyish-brown, lightly brown when dry. Blade elliptic-oblong, 16.5–20 (L) × 6–8.5 (W) cm; margin entire; apex cuspidate tip; bases attenuate. Petiole: striate, glabrous, 1.3–1.5 cm in length. ***Venation*** mid-rib wide, raised on both sides; pinnately veined, secondary venation eucamptodromous. Pairs of secondary nerves 10–11 pairs, raised on the underside. Tertiary veins sub-scalariform. ***Male and female inflorescences*** not seen. ***Peduncles*** up to 2–4 cm long and between 0.3 and 0.5 cm in diameter. ***Infructescence*** rachis diameter 0.4–0.5 cm. ***Acorn*** solitary along the rachis and spaced both in immature and mature stages. ***Cupule*** solitary and sessile, greenish-brown when fresh, mature cupules cup-shaped covering half of the nut, diameter 2.8–3.4 cm, cupule thickness 2.4–2.8 cm. thick-walled woody, cupule surface irregular, with a narrow ring of small denticulated plates around the rim, rest of cupule covered in distinct bullate protuberance gradually fusing into large tumour-like masses towards the base. Protuberances, specifically the rim, have resin burn marks with blackish shiny colour when dried. Immature cupules thin, cup-shaped covering 80% of the nut, covered in small protuberances ranging from relatively flat lines to bullate. ***Nut*** obovoid, length 1.9–2.3 cm, diameter 2.2–2.6 cm, sparsely tomentose around the basal scar, fawn-green when ripe, brownish-grey when dried, basal scar depressed, nut scar diameter 1.6–1.7 cm, thickness 0.3–0.4 cm. Resin leaking on the nuts. Apex flattened obtuse.

**Figure 1.**
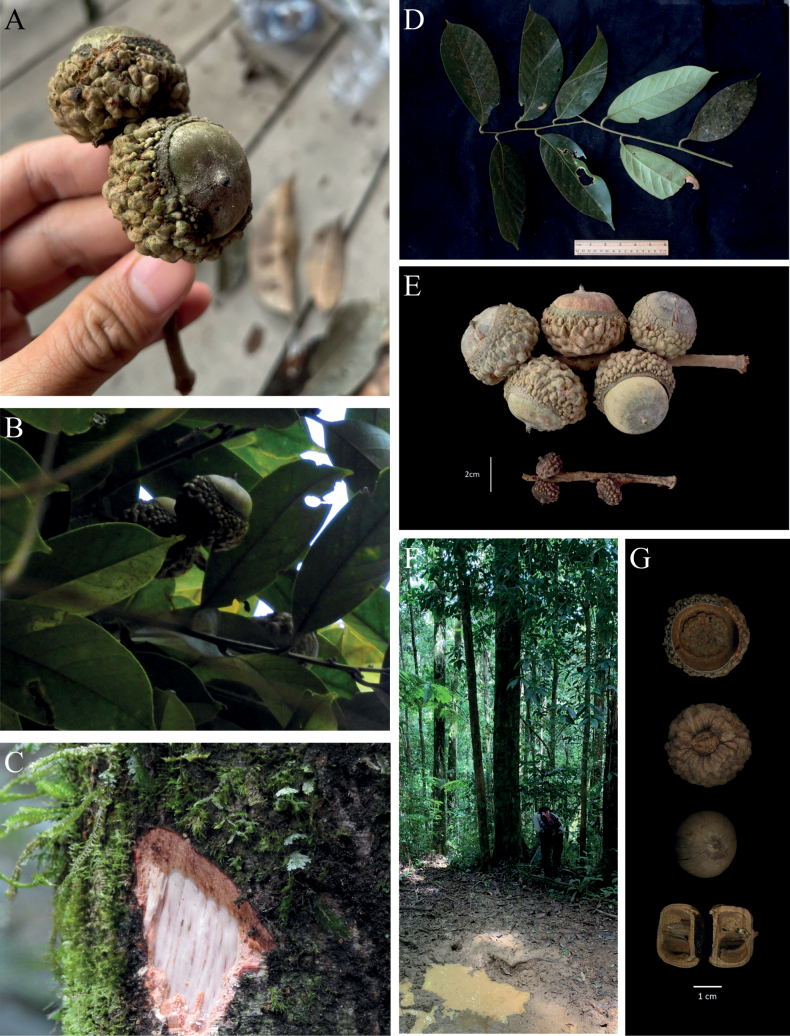
*Lithocarpustapanuliensis* Harapan, W.H.Tan, Nurainas & Strijk, sp. nov. **A** fresh fruits from field collection **B** fresh fruits in the canopy **C** bark and sapwood **D** fresh leaves **E** dried mature and immature infructescence **F** base of tree next to an animal wallow **G** cupule- bottom view, top view and nut bottom view and cross-section. Pictures by T.S. Harapan & T.A Febriamansyah, edited by W.H. Tan.

#### Phenology.

Fruiting was observed in February 2023 with fresh fruits recovered from the tree and from the ground.

**Figure 2.**
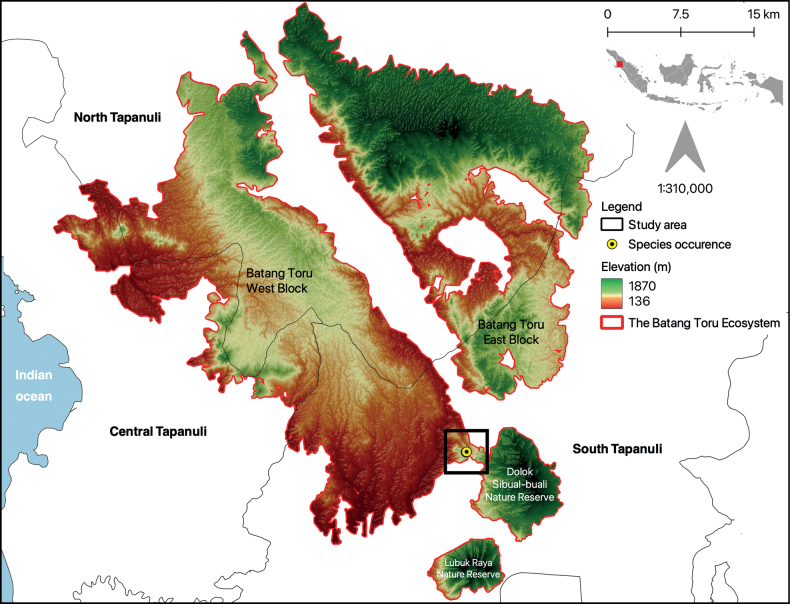
Distribution map of *Lithocarpustapanuliensis* sp. nov. in South Tapanuli, North Sumatra. The inset map shows the location of the sampling region on Sumatra Island, Indonesia. Elevation was obtained from SRTM (2023). Map by T.S. Harapan.

#### Distribution, habitat and ecology.

**Figure 3.**
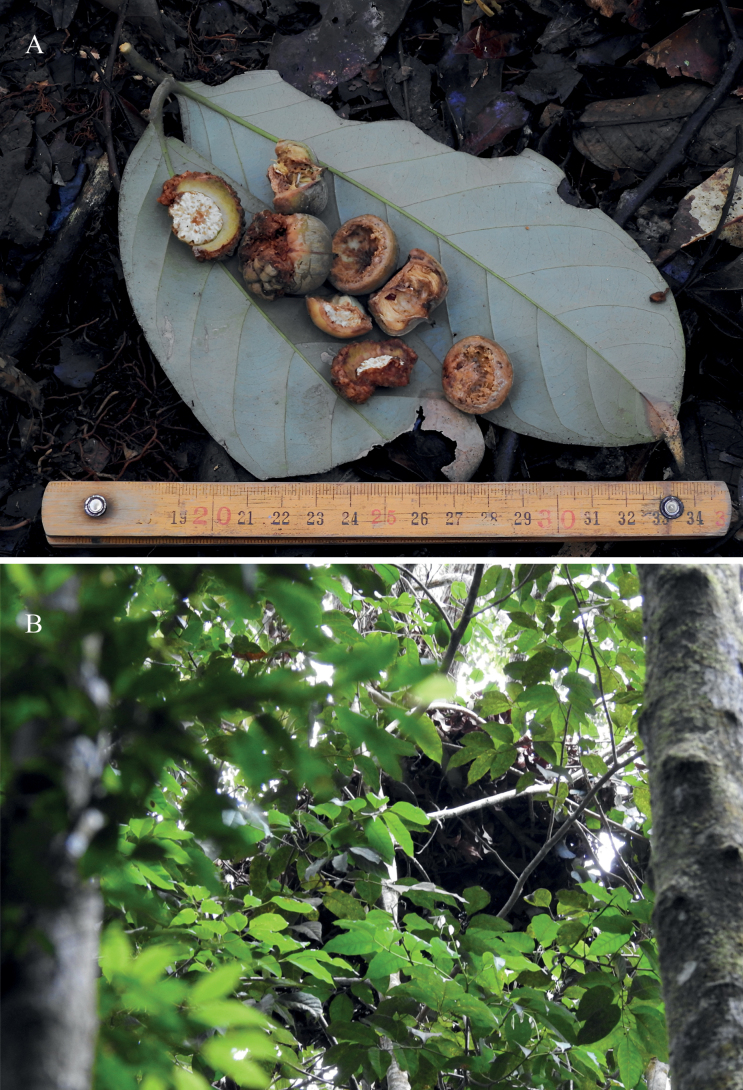
**A** Acorns consumed by orangutan **B** orangutan nest in a neighbouring tree. Pictures by T.S. Harapan & T.A Febriamansyah, edited by W.H. Tan.

During our fieldwork in Pilar Forest, a primary forest near the Bulu Mario District, we recorded two individuals of *Lithocarpustapanuliensis*. The lower-montane forest is characterised by the abundance of meranti gunung (*Shoreaplatyclados* Slooten ex Endert). Additional Fagaceae species were recorded, namely *Lithocarpusjavensis* Blume, *Quercusoidocarpa* Korth. and *Castanopsistungurrut* (Blume) A.DC. Interactions with Tapanuli orangutans were observed with a nest and remnants of consumed fruits were recorded near the tree (Fig. 3). Sipirok Regency precipitation typically varies during different sections of the year. Maximum monthly precipitation is 296.5 mm and the minimum monthly precipitation is 67 mm, with an average temperature around 28 °C (Badan Pusat Statistik 2023).

#### Vernacular name.

Hoteng (Tapanuli language).

#### Etymology.

The epithet is derived from its type locality, Tapanuli, South Tapanuli District, Sipirok Regency, North Sumatra Province, Indonesia.

#### Conservation status.

Using the guidelines established by the IUCN Red List (IUCN Standards and Petitions Committee 2022), we provide an initial conservation assessment of the species as Critically Endangered (B1ab(iii) + B2ab(iii), D), based on only two recorded individuals within Pilar Forest, its limited range and extensive habitat alteration and forest clearance in the immediate vicinity of the forest and throughout Sumatra. Pilar Forest does not have any legal protection or governance, but is immediately adjacent to Dolok Sibual-buali Nature Reserve. Both are part of the wider Batang Toru ecosystem landscape (150,000 ha; Fredriksson and Usher (2017)). Under this programme, the area is targeted for protection, ecosystem restoration and sustainable tourism development through a combination of NGOs and the State. More information and images are available on the species webpage (www.asianfagaceae.com/lithocarpus_tapanuliensis/) and GBIF.

## ﻿Discussion

There are an estimated 32 species of *Lithocarpus* documented in Sumatra (Soepadmo 1972; Purwaningsih and Polosakan 2016; POWO 2023; Strijk 2023). *Lithocarpustapanuliensis* sp. nov. is distinguished from other *Lithocarpus* in the region by its distinctly large acorn and a cupule covered in unique bullate protuberances and the distinct presence of a narrow ring of small denticulated plates around its rim. The possibilities of hybrids between stone oaks are rare because of the limited contact zone between closely-related taxa in our field sites (primary forest). A study in Mexico shows hybridisation of Quercus occurs in areas with high levels of disturbance (Tovar-Sánchez and Oyama 2004). Exposed and disturbed areas by humans may establish the hybrid zone (Howard et al. 1997; Tovar‐Sánchez and Oyama 2004) and enhance opportunities for contact and cross-pollination (Arnold et al. 1990; Klier et al. 1991).

The flora of Sumatra has garnered more interest over the last decade with a variety of plant species being described, like the iconic rafflesia (Susatya et al. 2017), enigmatic pitcher plants (Victoriano 2020), several begonias (Hughes et al. 2015) and a peculiar pipevine (Mustaqim and Arico 2022). To the authors knowledge, the discovery of *L.tapanuliensis* is the first new Sumatran *Lithocarpus* to be described in the last decade. This is a major contrast with other neighbouring botanical countries, in which a significant number of Fagaceae have been described in the last decade (e.g. Tan et al. (2023); Zhang et al. (2023)). The hotspots of *Lithocarpus* diversity are well-known to be in Indochina and NE Borneo, but the diversity of Sumatran Fagaceae has remained understudied. With further efforts in the future, the number of species confirmed for this island is expected to significantly increase.

During fieldwork, acorn remains of *L.tapanuliensis* and *C.tungurrut* consumed by Tapanuli orangutans were collected (Fig. 3A). It has been well documented that the acorns, leaves and bark of Fagaceae (i.e. *Lithocarpus*, *Castanopsis* and *Quercus*) are consumed by all three orangutan species (Russon et al. 2009; Kuze et al. 2011; Payne and Zainudin 2023). Fruit scarcity is a common occurrence in the seasonal Sundaic rainforest, due to the supra-annual fruiting cycle (within a period of 2–10 years) of many major tree families (e.g. Wong et al. (2005); Tan et al. (2021)). Although often overlooked, Sundaic Fagaceae are often a fall-back resource for many animals in periods of fruit scarcity, being one of the few families to have annual asynchronous fruiting (Araye et al. 2022). Besides the well-documented rodents, several large Asian megafaunal species, such as the Asian black bear (*Ursusthibetanus* Cuvier, 1823) and Malayan sun bear (*Helarctosmalayanus* Raffles, 1821) (Wong et al. 2002; Fredriksson et al. 2006; Steinmetz et al. 2013) often consume fruits of Fagaceae, especially in times of famine. The great migrations of the bearded pig (*Susbarbatus* Müller, 1838) across Borneo are dictated by the fruiting of several species, including those from Fagaceae (Lusking and Ke 2017). The importance of Fagaceae as a food supply for many species in times of famine should warrant greater protection for the family throughout its range in Southeast Asia as the loss of matured Fagaceae would have detrimental trophic effects.

An orangutan nest was also observed in a neighbouring tree to *L.tapanuliensis* (Fig. 3B). Orangutans are known to make their nest close to food sources and can be very selective of the tree species used. Species from families like Fagaceae and Dipterocarpaceae, with dense branching and tall, straight boles with thick diameters, are often favoured by orangutans for nest building, as they are relatively stable and provide good vantage points across the canopy (Kuswanda et al. 2020; Patana et al. 2021; Meylia and Mustari 2022). However, orangutans will avoid building nests in fruiting trees to avoid disturbance from other animals that also used the same resources (Sugardjito 1983; Van Casteren et al. 2012). The ecological interactions we recorded in the field further highlight the importance of Fagaceae to orangutans. Hence, we strongly recommend future conservation plans in the region to incorporate the family, not only for orangutans, but also for the myriad of other species that rely upon it for resources.

We provide an initial IUCN conservation assessment for *L.tapanuliensis* as Critically Endangered, as only two individuals were recorded in a small section of South Tapanuli, specifically in Pilar Forest (West Block of Batang Toru Ecosystem). The Batang Toru ecosystem suffers from habitat fragmentation and habitat loss due to massive infrastructure projects, such as mining, agroforestry plantations and hydropower in important corridor areas (e.g. Rahman et al. (2019)). Most of the Batang Toru area has been gazetted as protected forest, but some key forest areas are still unprotected (Fredriksson 2017; Sloan et al. 2018; Rahman et al. 2019). Recent infrastructure development near the study area could further fragment important habitats, increasing the risk of damaging the unique biodiversity found within this landscape. Conserving the remaining forest within the Batang Toru ecosystem is important to safeguard orangutans and many other mammals that depend on seasonal fruit production in highland and lowland forest areas of Batang Toru (Buij et al. 2002).

Our findings make an important contribution to the discovery of new Fagaceae species, highlighting the importance of preserving Indonesia’s unique habitats of the Batang Toru landscape. A comprehensive understanding of the species, along with further surveys and spatial distribution analysis, is crucial for protecting against potential extinction. Future strategies must focus on the long-term survival of the species through ex situ conservation in suitable habitats combined with in situ conservation efforts (Volis and Blecher 2010; Harapan et al. 2022).

## Supplementary Material

XML Treatment for Lithocarpus tapanuliensis

## ﻿Additional information

### Author ORCIDs

Try Surya Harapan https://orcid.org/0000-0002-6513-0012

Wei Harn Tan https://orcid.org/0000-0002-0971-7820

Syamsuardi https://orcid.org/0000-0001-8351-6528

Joeri Sergej Strijk https://orcid.org/0000-0003-1109-7015

### Data availability

All of the data that support the findings of this study are available in the main text.
